# Self-Powered Acceleration Sensor Based on Multilayer Suspension Structure and TPU-RTV Film for Vibration Monitoring

**DOI:** 10.3390/nano11102763

**Published:** 2021-10-18

**Authors:** Xiaotao Han, Qiyuan Zhang, Junbin Yu, Jinsha Song, Zhengyang Li, Haoran Cui, Jian He, Xiujian Chou, Jiliang Mu

**Affiliations:** Science and Technology on Electronic Test and Measurement Laboratory, School of Instrument and Electronics, North University of China, Taiyuan 030051, China; ihanxiaotao@163.com (X.H.); zqy-vv-xh@163.com (Q.Z.); yujunbin@nuc.edu.cn (J.Y.); jinsha2006daxue@163.com (J.S.); 18222586812@163.com (Z.L.); cuihaoran@nuc.edu.cn (H.C.); drhejian@nuc.edu.cn (J.H.); chouxiujian@nuc.edu.cn (X.C.)

**Keywords:** self-powered, acceleration sensor, doped-TPU, rough surface treatment, multilayer suspension

## Abstract

In this paper, we designed a triboelectric acceleration sensor with excellent multiple parameters. To more easily detect weak vibrations, the sensor was founded on a multilayer suspension structure. To effectively improve the electrical properties of the sensor, a surface roughening and internal doping friction film, which was refined with a room temperature vulcanized silicone rubber (RTV) and some thermoplastic polyurethanes (TPU) powder in a certain proportion, was integrated into the structure. It was found that the optimization of the RTV film increases the open circuit voltage and short circuit current of the triboelectric nanogenerator (TENG) by 223% and 227%, respectively. When the external vibration acceleration is less than 4 m/s^2^, the sensitivity and linearity are 1.996 V/(m/s^2^) and 0.999, respectively. Additionally, when it is in the range between 4 m/s^2^ and 15 m/s^2^, those are 23.082 V/(m/s^2^) and 0.975, respectively. Furthermore, the sensor was placed in a simulated truck vibration environment, and its self-powered monitoring ability validated by experiments in real time. The results show that the designed sensor has strong practical value in the field of monitoring mechanical vibration acceleration.

## 1. Introduction

Acceleration monitoring is an important source for people to obtain human–computer interaction information. However, the current monitoring equipment is relatively small and can only carry small-capacity batteries, which cannot meet the requirements of long-term real-time monitoring. Discarding used batteries causes environmental pollution, and the frequent charging or replacement of batteries is a tedious task for staff. In the attempts to lower dependence on traditional power sources, [1,2] triboelectric nanogenerator (TENG) have attracted great attention from governments and researchers all over the world [3]. TENG is extremely sensitive to environmental changes and can convert small amounts of energy in the environment into electrical energy [4,5,6]. Therefore, TENG-based acceleration sensors have also been greatly developed [7,8,9,10].

For self-powered sensors, there are four key parameters: sensitivity [11], linearity [12], range [13] and power generation [14]. At present, most self-powered acceleration sensors only one-sidedly pursue one or two parameters [15], which seriously affects their practicability. The composite self-powered acceleration sensor developed by Quan et al. [16] has a sensitivity as high as 143.2 V/(m/s^2^), but its applicable range is only 0.5–5 m/s^2^, which is very limited in application. The acceleration sensor developed by Zhang et al. [17] based on metal mercury droplet and nanofiber-networked polyvinylidene fluoride film, and the three-dimensional acceleration sensor developed by Pang et al. [18] have a large range (their ranges are 0–60 V/(m/s^2^) and 13–40 V/(m/s^2^), respectively) but the sensitivity of both does not exceed 0.3 V/(m/s^2^), resulting in lower accuracy of sensor monitoring. Wang et al. [14] invented a composite self-powered sensor for vibration and drop monitoring. The test range is 1–15 m/s^2^, and the sensitivity is about 1.53 V/(m/s^2^), but TENG’s power generation is only about 3 μW. Xiang et al. [11] invented a shock-resistant self-powered acceleration sensor, which has excellent linearity, but its monitoring range and sensitivity are only 0–6 m/s^2^ and 1.33 mV/(m/s^2^), respectively. In addition, some acceleration sensors only pay attention to the performance of the sensor, but ignore the protection of the device. If there is no direct protection for the sensitive unit of the TENG-based sensor, this would affect its versatility and useful lifespan [19]. Therefore, it is of great significance to ensure the various performances of the sensors are balanced and to improve the level of protection of the equipment. The TENG is available in four basic operating modes: vertical contact separation mode, horizontal sliding mode, single electrode mode, and independent layer mode. The vertical contact separation mode of the TENG is more suitable for mechanical vibration environments due to its high instantaneous power output and the ease of multilayer integration [20,21,22].

In this work, a multilayer suspended self-powered acceleration sensor (MSSAS) is designed. To effectively improve the electrical properties of the sensor, a surface roughening and internal doping friction film, which is refined with a room temperature vulcanized silicone rubber (RTV) and some thermoplastic polyurethanes (TPU) powder in a certain proportion, are integrated in the structure. The sensor is composed of a multilayer inner vibrating column and a multilayer shell, and the inner vibrating column is suspended in the shell by eight springs. The shell provides good protection for the internal vibration column, while the multilayer suspension structure improves the sensor’s perception of weak vibration and space utilization. Traditional acceleration sensors are composed of masses, dampers, elastic elements, and sensitive elements. In this design, the internal vibration column is used as the mass of the sensor, and the tension spring as the damper and elastic element. The TPU-RTV film and the conductive tape are used as the sensitive element. The magnitude of the acceleration of the sensor can be represented by the electrical signal of the TENG. The MSSAS can be used for the monitoring of vehicle’s vibration acceleration and the pre-detection of mechanical equipment. This study provides an in-depth theoretical investigation into the fabrication of a highly sensitive self-powered acceleration sensor.

## 2. Experimental

### 2.1. Materials

RTV’s shaping mold is made of polytetrafluoroethylene (PTFE), with an outer diameter of 50 mm and an inner groove diameter of 40 mm. The mesh range of the selected sandpaper is 80–600 meshes, and the thickness is 0.2 mm. Two specifications of metal tension springs (the wire diameter and length of the two types of tension springs are 0.3 mm and 15 mm, but the outer diameters are 4 mm and 3.5 mm, respectively) were purchased from Dingli Hardware Co., Ltd. (Hong Kong, China). The structure was made by 3D printing technology (raw material: 8200 resin). TPU powder (350 meshes), RTV liquid and its curing agent were purchased.

### 2.2. Preparation of the TPU-RTV Film

**Figure 1:**
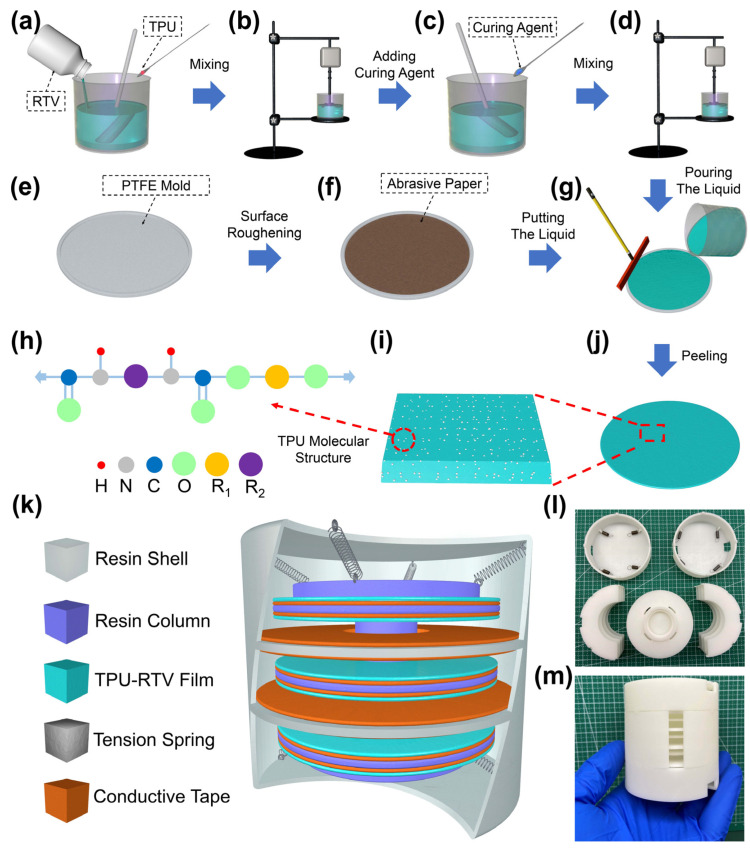
The production of the TPU-RTV film and the structure of the MSSAS. (**a**–**j**) The production process of the TPU-RTV film and the molecular structure of TPU. (**k**) The overall structure and material composition of the MSSAS. (**l**,**m**) Disassembly and assembly display of various parts of the MSSAS.

The manufacturing process of the TPU-RTV film is shown in Figure 1a–h. First, an appropriate amount of liquid RTV is poured into a beaker, then a certain mass ratio of TPU powder is added to the beaker. During the addition, the mixed solution is continuously stirred with a glass rod. After the addition, the mixed solution is placed on a mechanical stirrer and stirred at a speed of 500 r/min for 5 h. After stirring, the curing agent is added to the mixed solution according to a mass ratio of 51:1, and stirred again for 20 min. The sandpaper is cut into the size of a PTFE-Mold and attached to the bottom of the mold. Then, the stirred mixed solution is poured into the mold, and the surface of the mold is smoothed with a spatula. Finally, PTFE-Mold is placed on a horizontal table for 24 h, and then the TPU-RTV film with a certain TPU doping concentration can be peeled off.

### 2.3. Assembly of the MSSAS

The overall structure of the MSSAS is shown in Figure 1k. It consists of a cylindrical shell (radius of 36 mm, high of 74.5 mm) and an internal vibration column (radius of 30 mm, high of 40 mm). The inner wall of the shell and the outer wall of the vibration module were both grown with multiple thin interlayers and the interlayer spacing, width, and thickness are 9.5 mm, 13 mm, and 1.5 mm, respectively. The shell and the internal vibration module are interwoven with each other through the interlayer, and they are connected by eight tension springs on the upper and lower sides. The positive friction material is the conductive tape, which is attached to both sides of the interlayer and the negative friction material is the TPU-RTV film, which is attached to the conductive tape in the interlayer of the vibrating column. Due to the gravity of the internal vibration module, the elastic force of the top spring is slightly greater than that of the bottom spring, ensuring that the vibration module is in suspension. The physical object of the MSSAS and the disassembly of each part are shown in Figure 1l,m.

## 3. Results and Discussion

### 3.1. Characterization and Operating Principle of the MSSAS

**Figure 2:**
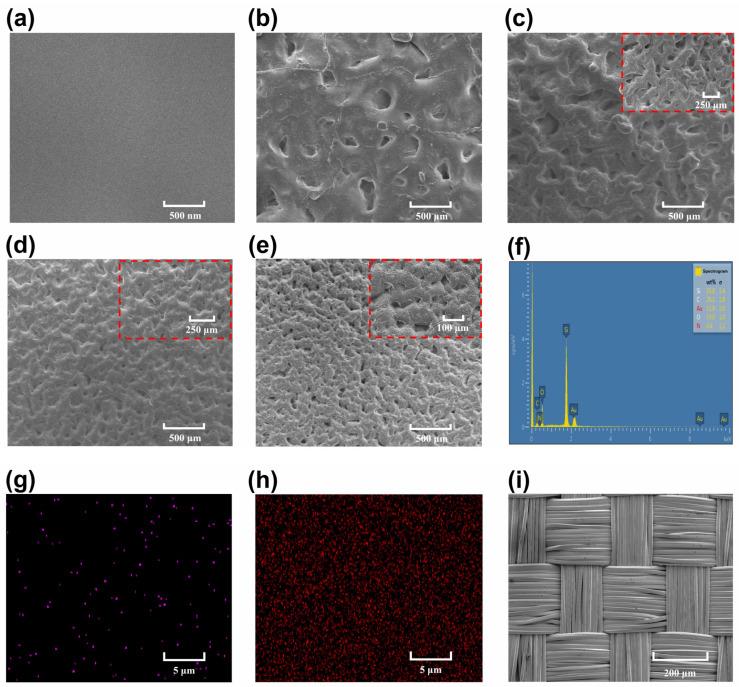
Surface observation of the TPU-RTV film and the conductive tape. (**a**–**e**) SEM image of the TPU-RTV film with different surface roughness (from 0 mesh to 600 meshes). (**f**–**h**) EDS image of the TPU-RTV film (the doping ratio of TPU is 3%). (**i**) SEM image of the conductive tape.

The TPU-RTV films with different roughness were scanned by scanning electron microscope (SEM, SU8020, Hitachi, Tokyo, Japan) (Figure 2a–e). It can be seen that there are obvious irregular holes on the surface of the TPU-RTV film. With the increase in sandpaper meshes, the number of holes on the film surface gradually increases, and the size of the holes becomes smaller and smaller. The film with 3% TPU doping is analyzed by an energy dispersive spectrometer (EDS) (Figure 2f). TPU mainly consists of four elements: C, H, O, and N, while RTV itself contains a large amount of C and O. In addition, EDS cannot detect H, so the N is used to characterize the doping of TPU. The proportion of TPU doping is only 3%, and the proportion of N detected is relatively small. By observing the scanned image of the EDS, it can be found that the N is evenly distributed on the surface of the film (Figure 2g). This shows that TPU is uniformly doped inside the film. Figure 2h shows the distribution of Si on the surface of the film. Figure 2i shows the SEM image of the surface of the conductive adhesive tape, which is in a woven structure. In addition, the films were analysed by XPS and FT-IR. Figure S1a shows the complete XPS spectrum of the film before and after TPU doping. The characteristic peaks of the films including Si 2p, C 1s, O 1s peaks were all identified. The three characteristic peaks are located at 100.78 eV, 283.48 eV, and 530.88 eV. In addition, the doped TPU film detected a characteristic peak of N1s at 398.40 eV. Since the proportion of N in the TPU molecule is low, the characteristic peak of N is relatively short. N1s exists in the TPU chain and forms an N-H group with H. This conclusion was verified in FT-IR. Figure S1b shows the FT-IR spectra of the TPU-RTV film. TPU-specific groups were examined, such as N-H, which is mainly distributed at 3600 cm^−1^. The N-H group is an important part of the hard and soft segments of the TPU molecule, which gives the TPU stretchability and fracture resistance, and can also improve the physical properties of the film after doping [23].

**Figure 3:**
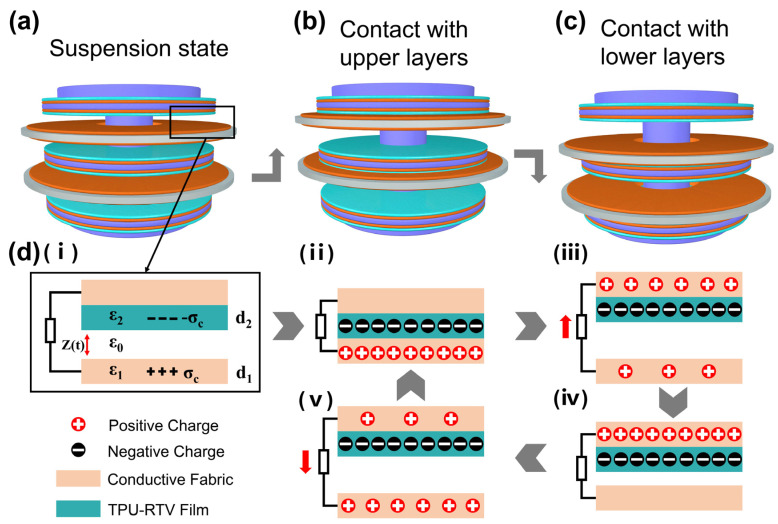
Working principle of the MSSAS. (**a**–**c**) The MSSAS in different states of motion. (**d**) Principle of charges transfer, **d**(**i**–**v**) The MSSAS working state cycle.

As shown in Figure 3a–c, the working mechanism of the MSSAS is contact separation mode TENG. Assuming that the dielectric constant of the vacuum environment is *ε*_0_. *ε*_1_ and *ε*_2_, respectively, represent the dielectric constant of the TPU-RTV film and the conductive tape. The distance between the TPU-RTV film and the conductive tape is a function *Z(t)* of *t*, and the thickness of the two is *d*_1_ and *d*_2_. Due to the triboelectric effect [24], the surfaces of the two friction layers generate equal amounts of charges with opposite polarity, and the surface charge densities are *σ_c_* and *−σ_c_*. A friction electric field will be formed between the friction layers of opposite polarity, and the field strength $\sigma_{I}(Z,t)$ is a function of the distance between the friction layers (Figure 3d(i)).

The field strength inside the TPU-RTV film and the conductive tape [25]:

(1) $$E_{Z}=\sigma_{I}(Z,t)/\varepsilon_{1}$$

(2) $$E_{Z}=\sigma_{I}(Z,t)/\varepsilon_{2}$$

Field strength between the TPU-RTV film and the conductive tape:

(3) $$E_{Z}=(\sigma_{I}(Z,t)-\sigma_{c})/\varepsilon_{0}$$

The voltage between the positive and negative electrodes:

(4) $$V=\sigma_{I}(Z,t)[d_{1}/\varepsilon_{1}+d_{2}/\varepsilon_{2}]+Z[\sigma_{I}(Z,t)-\sigma_{c}]/\varepsilon_{0}$$

When the circuit is short-circuited, the field strength of the friction electric field:

(5) $$V=0,\sigma_{I}(Z,t)=\frac{Z\sigma_{c}}{d_{1}\varepsilon_{0}/\varepsilon_{1}+d_{2}\varepsilon_{0}/\varepsilon_{2}+Z}$$

The displacement current density can be obtained as:

(6) $$J_{D}=\frac{\partial \sigma_{I}(Z,t)}{\partial t}=\sigma_{c}\frac{dZ}{dt}\frac{d_{1}\varepsilon_{0}/\varepsilon_{1}+d_{2}\varepsilon_{0}/\varepsilon_{2}}{{\left[d_{1}\varepsilon_{0}/\varepsilon_{1}+d_{2}\varepsilon_{0}/\varepsilon_{2}+Z\right]}^{2}}$$

According to Equation (6), the displacement current density is proportional to the contact and separation speed of the two friction layers. The output of TENG is proportional to the magnitude of acceleration; therefore, the output of TENG can be used to characterize the magnitude of acceleration.

Figure 3d(i–v) demonstrates a working cycle of the MSSAS [26,27]. In the static state, the internal vibrating column is suspended in the MSSAS under the action of eight springs. There is no contact with the interlayer of the inner wall of the shell at this time. When vibration occurs from the outside, the shell of MSSAS will move upward (or downward) instantly. Due to the buffering and inertial action of the spring, the vibration module will not move with the shell for the first time. The shell and the vibration module will move relative to each other. When the TPU-RTV film is in contact with the conductive tape, the charges will accumulate on the surface of the two materials (Figure 3d(ii)) [28]. Then, the MSSAS’s shell moves downward (upward), and the vibration module moves upward (downward) under the traction force of springs. The TPU-RTV film and the conductive tape change from contact to separation. Under the action of the internal electric field, electrons flow from the upper electrode to the lower electrode through the outer wire to form a current (Figure 3d(iii)). When the shell rises (falls) again, the TPU-RTV film and the conductive tape will contact again, forming a current in the opposite direction to that of separation (Figure 3d(iv,v)) [29].

### 3.2. Properties of Films with Different Doping and Roughness

In order to improve the sensitivity and self-powered ability of the MSSAS, the RTV film is improved by doping TPU and changing the surface roughness.

**Figure 4:**
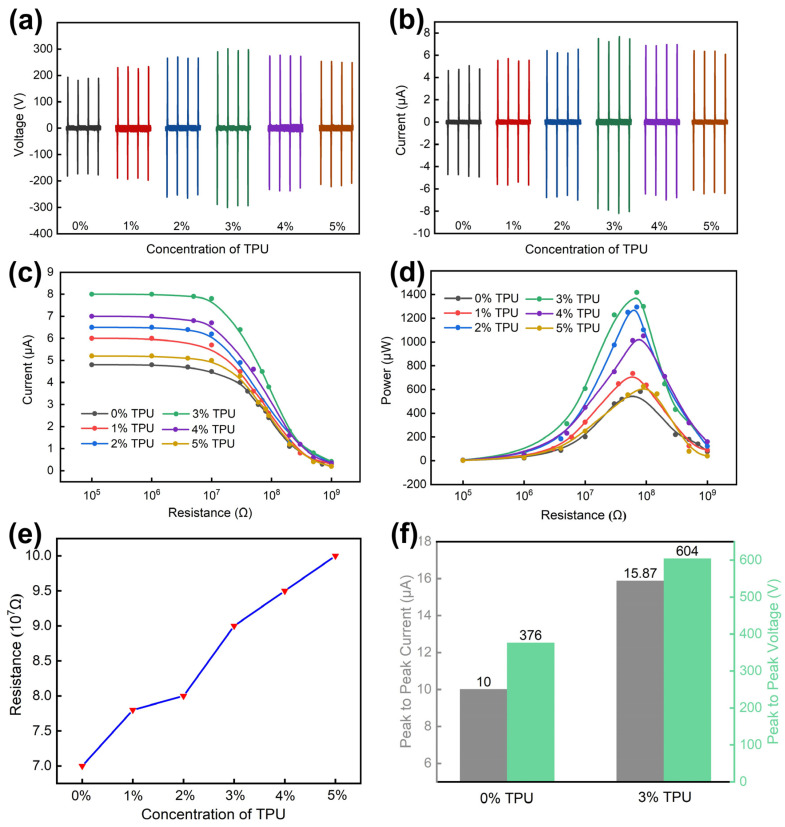
The effect of TPU doping concentration on the output performance of the film. (**a**,**b**) The Voc and the Isc of TENG under different doping concentrations (from 0 wt% to 5 wt%). (**c**,**d**) After connecting different resistors in series, the influence of TPU doping concentration on the electrical properties of TENG. (**e**) The influence of TPU doping concentration on the internal resistance of the film. (**f**) Comparison of electrical properties of films with TPU doping concentration of 0% and 3%.

Figure 4 shows the effect of doping TPU on the electrical properties of the RTV film. The thickness and diameter of the TPU-RTV film are 0.7 mm and 40 mm, respectively, and the working frequency of connecting rod machine is 1.5 Hz. According to Figure 4a,b, as the doping concentration of TPU increases, the open circuit voltage (Voc) and short circuit current (Isc) of TENG both increase first and then decrease. When the doping ratio of TPU is 3%, the electrical performance of TENG reaches its best state. The TENG’s peak-to-peak value of the Voc is 604 V (Figure 4f), and the TENG’s peak-to-peak value of the Isc is 15.88 μA. Additionally, they are increased by 60.64% and 58.77%, respectively, compared with the undoped RTV film. TENG is connected in series with resistors of different resistance (from 10 Ω to 10 MΩ). By measuring the current of the series circuit (*I_TENG_*), according to the equation: $P_{TENG}=I_{TENG}{}^{2}R_{TENG}$ (*R_TENG_* is the external resistor), the output power of TENG (*P_TENG_*) is obtained. As shown in Figure 4c,d, when the TPU doping concentration is constant, as the series resistance increases, the current of the series circuit decreases, but the output power of TENG tends to increase first and then decrease. When the TPU doping concentration of the film is 3% and the external resistance is 9 × 10^7^ Ω, the output power of TENG reaches the maximum value of 1417.5 μW. The main reason for doping TPU to increase the output of TENG is that TPU powder has a higher dielectric constant. RTV’s dielectric constant is in the range of 3.5~3.6 [30], and the dielectric constant of TPU is in the range of 6~7 [31] (room temperature, 50 Hz). The maximum transferred charge density *σ′* can be expressed as Equation (7) [32,33].

(7) σ′=σddgapdgap+dcoatingεcoating

where *σ_d_*, *d_gap_*, *d_coating_*, and *ε_coating_* are the triboelectric charge densities at the equilibrium state, gap distance, thickness of the TPU-RTV film, and dielectric constant of the TPU-RTV film, respectively. The equation shows that the maximum transfer charge density increased as the dielectric constant increased. Therefore, the dielectric constant of the RTV film can be improved by doping TPU powder. As the doping concentration of TPU increases, the output power of TENG shows an upward trend. However, the increase in TPU doping concentration will increase the internal resistance of the film (Figure 4e). When the doping concentration of TPU is 0%, the internal resistance of the film is about 7 × 10^7^ Ω and when the doping concentration of TPU is 5%, the internal resistance of the film is 10 × 10^7^ Ω. Assume that the internal resistance of the film and the external load resistance are *r* and *R*, respectively, and the electromotive force of TENG is *E*.

The output power of TENG:

(8) $$P_{o}=\frac{E^{2}}{\frac{{\left(R-r\right)}^{2}}{R}+4r}$$

When *R* is equal to *r*, the output power reaches the maximum value:

(9) $$P_{\max}=\frac{E^{2}}{4r}$$

Therefore, when the doping concentration of TPU is too high, the internal resistance of the film will increase and the output power of TENG will decrease. Considering the positive and negative effects of doping concentration, the doping concentration of TPU will have an optimal ratio to maximize the output power of TENG. After experimental verification, the optimal doping concentration of TPU is 3%.

**Figure 5:**
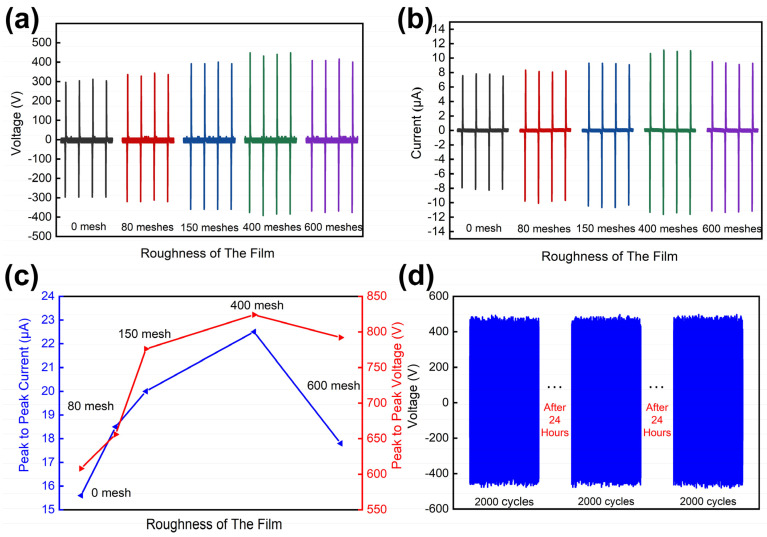
The influence of film’s surface roughness on the electrical properties of TENG. (**a,b**) The Voc and Isc of TENG under different surface roughness. (**c**) TENG’s peak-to-peak value of Voc and Isc under different surface roughness. (**d**) Stability test of the TPU-RTV film.

To verify the influence of the film’s surface roughness on the electrical properties of TENG, the films with different roughness and the conductive tapes were placed on the connecting rod machine with a working frequency of 1.5 Hz. Additionally, the doping concentration of TPU, the thickness and diameter of the film were uniformly fixed at 3%, 0.7 mm and 40 mm, respectively. The test results are shown in Figure 5. It is clear that the output performance of TPU-RTV films with surface roughness treatment is improved compared to that before treatment. This is because the roughness of the film surface increases the effective contact area of the two friction layers, which results in an increase in the electrical properties of the TENG [34,35]. According to Figure 5a,b, it can be seen that as the surface mesh of the film increased, the output performance of TENG first increased and then decreased. When the surface roughness of the TPU-RTV film was 400 meshes, the Voc and Isc of TENG reached the maximum, and the peak-to-peak values are 840 V and 22.73 μA, respectively (Figure 5c). Compared with the unroughened film, the peak-to-peak value of Voc of TENG increased by 38.16%, and the peak-to-peak value of Isc is increased by 41.53%. It was thereby experimentally verified that when the roughness of the film is 400 meshes, the output performance of TENG is the best and the effective contact area of the two friction layers is the largest [36].

The stability of the TPU-RTV film was also tested (the circular film was 0.7 mm in thickness, 40 mm in diameter, 400 meshes in roughness and the doping concentration of TPU was 3%). The film and conductive tape were placed on the connecting rod machine with a working frequency of 3 Hz. TENG’s Voc data were recorded in one group every 24 h, and each group of data contained 2000 contact separation cycles (Figure 5d). By observing the three sets of test results of TENG, it was found that the Voc of TENG was stable at −460–480 V, which indicates that the TPU-RTV film has excellent stability.

### 3.3. Linear Regression Analysis of the MSSAS

**Figure 6:**
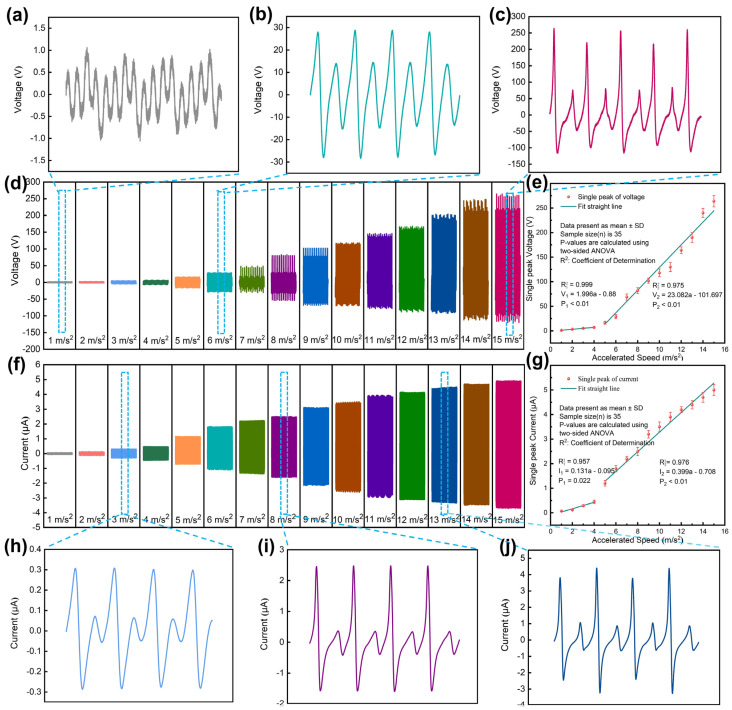
Electrical characteristics of the MSSAS. (**a**–**c**) Amplified display chart of some Voc signals of the MSSAS (1 m/s^2^, 6 m/s^2^, and 15 m/s^2^). (**d**,**f**) The Voc and Isc of the MSSAS under different accelerations (from 1 m/s^2^ to 15 m/s^2^). (**e**,**g**) Linear regression lines of single-peak voltage signals and current signals on vibration acceleration. (**h**–**j**) Amplified display chart of some Isc signals of the MSSAS (3 m/s^2^, 8 m/s^2^, and 13 m/s^2^).

The TPU-RTV film and conductive tape were equipped in the MSSAS. In order to verify the linearity and sensitivity of the MSSAS under different accelerations, the MSSAS was placed on a shaker, and the electrical signals of the MSSAS in a vibration environment of 1–15 m/s^2^ were tested in turn. The Voc of the MSSAS is shown in Figure 6d. According to its changing trend, the voltage response was divided into two regions of 1–4 m/s^2^ and 4–15 m/s^2^, respectively. SPSS Statistics 25 Software was used to analyze the sensitivity and linearity. The linear regression equations of single peak voltage (*V*_1_, *V*_2_) of the MSSAS are: $V_{1}=1.996a-0.880$ and $V_{2}=23.082a-101.679$, respectively (*a* is the vibration acceleration of the machine) (Figure 6e). The sensitivity of the MSSAS can be obtained as: 1.996 V/(m/s^2^), 23.082 V/(m/s^2^), and the voltage linearity is 0.999 and 0.975, respectively. The four cycles of Voc for the MSSAS in a vibration environment of 1 m/s^2^, 6 m/s^2^ and 15 m/s^2^ are shown in Figure 6a–c, respectively. When the external acceleration was 1 m/s^2^, the internal vibration module of the MSSAS performed regular high-frequency fine vibration. There were four contact separations with different amplitudes in a complete cycle, but its voltage response was stable between −1 V and 1 V. When the external acceleration was 6 m/s^2^, the internal vibration module performed simple harmonic vibration. It contacted and separated from the upper and lower interlayers of the shell once in each cycle. Since the tension of the top and bottom springs of the vibration module are different, there will be one high voltage response and one low voltage response in one cycle. When the external acceleration was 15 m/s^2^, the relative acceleration of the shell and the vibration module further increased, and the voltage response increased accordingly. The current response of the MSSAS under different accelerations is shown in Figure 6f. The linear regression equations of single peak current (*I*_1_, *I*_2_) of the MSSAS are: $I_{1}=0.131a-0.095$ and $I_{2}=0.399a-0.708$, respectively (*a* is the vibration acceleration of the machine) (Figure 6g). The sensitivity of the MSSAS in the two regions is 0.131 μA/(m/s^2^) and 0.399 μA/(m/s^2^), and the linearity is 0.957 and 0.976, respectively. Figure 6h–j shows the four cycles of Isc for the MSSAS in vibration environments of 3 m/s^2^, 8 m/s^2^ and 13 m/s^2^, respectively. The different works have unique advantages and application scenarios in terms of energy harvesting or the particular performance of a sensor [37,38,39,40,41]. In this paper, the MSSAS has a good balance of sensitivity, linearity and range, as well as energy harvesting capability (Table S1).

### 3.4. Application Analysis

With the development of fresh food e-commerce and the transformation of new retail, the logistics and transportation industry of fruits and vegetables has entered a period of rapid development. Due to truck vibration, more than 80% of various types of fruits may be mechanically damaged during truck transportation, which is also one of the main factors for the loss of fruits and vegetables after picking [42]. Therefore, real-time monitoring and early warning of the vibration acceleration of the truck body is very necessary. According to the research, when the vibration acceleration of the truck body exceeds 7 m/s^2^, even thick-skinned fruits, such as watermelon and olives, will be severely damaged [43]. The MSSAS has good linearity and sensitivity over the range of transport vibration acceleration of fruits. It can monitor the vibration of the truck in real time, and thus determine whether the fruit will be damaged.

**Figure 7:**
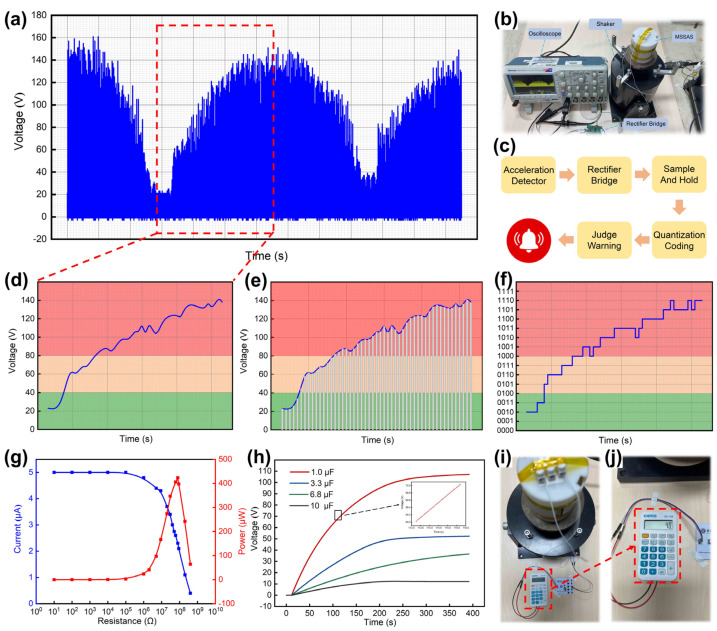
The MSSAS monitors mechanical vibration acceleration and self-powered performance. (**a**) Real-time monitoring of the shaker by the MSSAS. (**b**) Test environment. (**c**–**f**) Processing of real-time monitoring signals. (**g**) The Isc and output power of the MSSAS with series resistors of different resistance. (**h**) Different size capacitors are charged by the MSSAS (the capacitances are 1 μF, 3.3 μF, 6.8 μF, 10 μF). (**i**,**j**) The small electronic calculator is powered by the MSSAS.

A shaker was used to simulate the vibration of the truck, constantly changing the vibration acceleration of the shaker. Video S1 and Figure 7a show the voltage response of the MSSAS in real time after rectification. Suppose that the safe vibration acceleration of a certain fruit is $a<6\;m/s^{2}$, and the early warning vibration acceleration is $6\;m/s^{2}\leq a\leq 8\;m/s^{2}$, and the dangerous vibration acceleration is $8\;m/s^{2}<a$. Figure 7d shows the envelope of a portion of the voltage response signal. Then, the voltage signal was sampled at equal time intervals and stored for a short period of time after each sample was obtained (Figure 7e). The analog signal that changed continuously in time was transformed into a pulse signal. The minimum quantization unit was set to 10 V, and the measured voltage value could be normalized to an integer multiple of 10 V, and then the normalized result could be expressed by binary code (Figure 7f). According to the linear regression equation of single-peak voltage and vibration acceleration, the voltage value can be used to characterize the vibration acceleration so that the magnitude of the vibration acceleration can be obtained by observing the voltage code value. In this way, the safe voltage code range is F<0100, and the warning voltage code is 0100≤F≤1000, and the dangerous voltage code is 1000<F.

To verify the self-powered performance of the MSSAS, it was connected in series with different resistance (*R_M_*) and the current (*I_M_*) in the circuit was measured. According to the equation: $P_{M}=I_{M}^{2}R_{M}$, the output power of the MSSAS (*P_M_*) was calculated. The test result is shown in Figure 7g. When the external resistance is 10^8^ Ω, the maximum output power of the MSSAS can reach 423.2 μW. Figure 7h shows four different sizes of capacitors being charged by the MSSAS. A 1 μF capacitor was charged from 0 V to 107.1 V in 400 s, and a 10 μF capacitor was charged from 0 V to 12.1 V in the same amount of time. In addition, the MSSAS was used to power an electronic calculator to enable mathematical calculations (Figure 7i,j) (Video S2).

## 4. Conclusions

In summary, a sensor with a novel structure and a modified friction material was designed. The sensor was composed of a multilayered protective shell and an internal vibrating column. The shell and the vibrating column were staggered and connected by eight tension springs, which effectively enhanced the perception of vibration and space utilization. The conductive tape and RTV film were selected as the friction layers. The RTV films were optimized by surface roughening treatment and blended with 3% mass of TPU powder. This resulted in a 223% and 227% increase in the peak-to-peak values of Voc and Isc of TENG, respectively, at 1.5 Hz of external impact. The improvement in TENG’s electrical performance also effectively improved the sensitivity and self-powered performance of the sensor. The sensor acceleration monitoring range was 1–15 m/s^2^. When the external vibration acceleration was 1–4 m/s^2^, the sensitivity and linearity of the MSSAS were 1.996 V/(m/s^2^) and 0.999, respectively. Additionally, when the external vibration acceleration was 4–15 m/s^2^, the sensitivity and linearity of the MSSAS were 23.082 V/(m/s^2^) and 0.975, respectively. In addition, the MSSAS also performed real-time monitoring of the shaker acceleration. This test verified the applicability and reliability of the MSSAS in mechanical vibration monitoring and demonstrated the feasibility of the TENG-based sensor. TENG-based sensors have great research value and development potential under the condition of scarce resources and serious environmental pollution by fossil fuels.

## 5. Experimental Section

### 5.1. Measurement System

Scanning electron microscope (SEM) (Hitachi SU8020, Chiyoda-ku, Japan) was used to observe the surface morphology of the TPU-RTV film, and the element distribution was qualitatively tested with energy dispersive spectrometer (EDS). An oscilloscope (MSO2024B, Tektronix, Beaverton, OR, USA) and an electrometer (6514, Keithley, Cleveland, OH, USA) were used to measure the output voltage and output current of the TENG part and the MSSAS. Additionally, a shaker (JZQ-200) was used to simulate applied force.

### 5.2. Statistical Analysis

The voltage and current data were preprocessed to clean up abnormal data. The detection of outliers used the method of mean square error. If the voltage or current data of a certain point exceed three times the standard deviation, then these points were defined as outliers. If there were missing values or outliers in one set of data, then this set of data was discarded. The single peak voltage-acceleration (or single peak current-acceleration) data were subjected to linear regression analysis using SPSS Statistics 25 Software. The sample size was thirty-five. *p*-values were calculated using two-sided ANOVA. The Origin 2018 Software was also used for statistical analysis.

## Supplementary Materials

The following are available online at https://www.mdpi.com/article/10.3390/nano11102763/s1, Figure S1: (a) Material properties of the films tested by XPS. (b) Material properties of doped films tested by FT-IR. Table S1: Comparison of vibration energy harvesting and self-powered sensors. Video S1: Real-time monitoring of a shaker by the MSSAS. Video S2: The MSSAS powers a small calculator.
