# Supplementary material for: Self-Powered Acceleration Sensor Based on Multilayer Suspension Structure and TPU-RTV Film for Vibration Monitoring
Source: Nanomaterials (Basel). 2021 Oct 18;11(10):2763. doi: 10.3390/nano11102763 (PMC8538356; doi:10.3390/nano11102763)
Supplement: Supplementary file 1 [file nanomaterials-11-02763-s001.zip › nanomaterials-1375732-SI.pdf]

Article

Self-Powered Acceleration Sensor Based on Multilayer Suspension Structure and TPU-RTV Film for Vibration Monitoring

Xiaotao Han, Qiyuan Zhang, Junbin Yu, Jinsha Song, Zhengyang Li, Haoran Cui, Jian He, Xiujian Chou and Jiliang Mu *

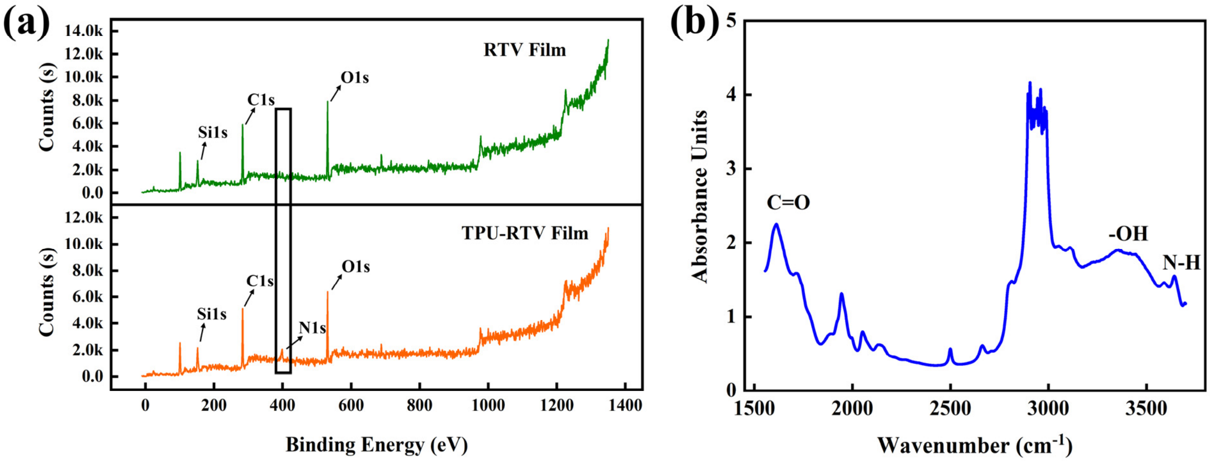

Figure S1. (a) Material properties of the films tested by XPS. (b) Material properties of doped films tested by FT-IR.

Table S1. Comparison of vibration energy harvesting and self-powered sensors

Sensitivity (V/(m/s²))	Linearity	Range (m/s²)	Output voltage (V)	References
N.A.	N.A.	1.07 ~ 1.25	3.1	[1]
143.2	N.A.	0.5 ~ 5	245	[2]
17.64	0.99	0 ~ 1.76×10^5	35	[3]
0.26	N.A.	0 ~ 60	15.5	[4]
0.29	N.A.	13 ~ 40	20	[5]
1.53	N.A.	1 ~ 15	16	[6]
1.33	N.A.	0 ~ 6	0.01	[7]
N.A.	N.A.	N.A.	316	[8]
N.A.	N.A.	N.A.	1	[9]
N.A.	N.A.	N.A.	150	[10]
N.A.	N.A.	N.A.	8.2	[11]
N.A.	0.99	N.A.	0.4	[12]
23.08	0.98	1 ~ 15	250	This work

1. Zhao, X.; Wei, G.; Li, X.; Qin, Y.; Xu, D.; Tang, W.; Yin, H.; Wei, X.; Jia, L., Self-powered triboelectric nano vibration accelerometer based wireless sensor system for railway state health monitoring. *Nano Energy* **2017**,

34, 549-555. [CrossRef]
2. Quan, T.; Wang, Z. L.; Yang, Y., A Shared-Electrode-Based Hybridized Electromagnetic-Triboelectric Nanogenerator. *ACS Appl Mater Interfaces* 2016, *8* (30), 19573-19578. [CrossRef]
3. Dai, K.; Wang, X.; Yi, F.; Jiang, C.; Li, R.; You, Z., Triboelectric nanogenerators as self-powered acceleration sensor under high-g impact. Nano Energy 2018, 45, 84-93. [CrossRef]
4. Zhang, B.; Zhang, L.; Deng, W.; Jin, L.; Chun, F.; Pan, H.; Gu, B.; Zhang, H.; Lv, Z.; Yang, W.; Wang, Z. L., Self-Powered Acceleration Sensor Based on Liquid Metal Triboelectric Nanogenerator for Vibration Monitoring. *ACS Nano* **2017**, *11* (7), 7440-7446. [CrossRef]
5. Pang, Y. K.; Li, X. H.; Chen, M. X.; Han, C. B.; Zhang, C.; Wang, Z. L., Triboelectric Nanogenerators as a Self-Powered 3D Acceleration Sensor. *ACS Appl Mater Interfaces* **2015**, *7* (34), 19076-19082. [CrossRef]
6. Wang, L.; He, T.; Zhang, Z.; Zhao, L.; Lee, C.; Luo, G.; Mao, Q.; Yang, P.; Lin, Q.; Li, X.; Maeda, R.; Jiang, Z., Self-sustained autonomous wireless sensing based on a hybridized TENG and PEG vibration mechanism. *Nano Energy* **2021**, *80*, 105555. [CrossRef]
7. Xiang, C.; Liu, C.; Hao, C.; Wang, Z.; Che, L.; Zhou, X., A self-powered acceleration sensor with flexible materials based on triboelectric effect. *Nano Energy* **2017**, *31*, 469-477. [CrossRef]
8. Qian, J.; Kim, D.; Lee, D., On-vehicle triboelectric nanogenerator enabled self-powered sensor for tire pressure monitoring. *Nano Energy* **2018**, *49*, 126-136.[CrossRef]
8. Askari, H.; Saadatnia, Z.; Khajepour, A.; Khamesee, M.; Zu, J., A Triboelectric Self-Powered Sensor for Tire Condition Monitoring: Concept, Design, Fabrication, and Experiments. *Advanced Engineering Materials* 2017, 19, 1700318. [CrossRef]
10. Wu, W.; Cao, X.; Zou, J.; Ma, Y.; Wu, X.; Sun, C.; Li, M.; Wang, N.; Wang, Z.; Zhang, L., Triboelectric Nanogenerator Boosts Smart Green Tires. *Advanced Functional Materials* **2018**, *29*, 1806331.[CrossRef]
11. Wang, X.; Niu, S.; Yi, F.; Yin, Y.; Hao, C.; Dai, K.; Zhang, Y.; You, Z.;Wang, Z. L., Harvesting Ambient Vibration Energy over a Wide Frequency Range for Self-Powered Electronics. *ACS Nano* **2017**, 11, 1728-1735.[CrossRef]
12. Maharjan, P.; Bhatta, T.; Cho, H.; Hui, X,; Park, C.; Yoon, S.; Salauddin, M.; Rahman, M.; Rana, S.; Park, J., A Fully Functional Universal Self‐Chargeable Power Module for Portable/Wearable Electronics and Self‐Powered IoT Applications *Advanced Energy Materials* **2020** ,10, 2002782 [CrossRef]

Video S1. Real-time monitoring of a shaker by the MSSAS.

Video S2. The MSSAS powers a small calculator.
